# Neoadjuvant chemotherapy versus long-course neoadjuvant chemoradiotherapy for locally advanced rectal cancer: a systematic review and meta-analysis of 5,168 cases

**DOI:** 10.3389/fonc.2025.1542885

**Published:** 2025-06-26

**Authors:** Wenjie Zhou, Xueting Wang, Jie Dan, Minjie Zhu, Qian Liao, Ke Liu, Jiangpeng Li, Xianhong Jiang, Yonghong Wang

**Affiliations:** ^1^ Department of Gastrointestinal Surgery, The People’s Hospital of Leshan, Leshan, China; ^2^ Department of Scientific Research and Teaching, The People’s Hospital of Leshan, Leshan, China

**Keywords:** locally advanced rectal cancer, neoadjuvant, chemotherapy, chemoradiotherapy, systematic review, meta-analysis

## Abstract

**Background:**

Long-course neoadjuvant chemoradiotherapy (Lc-NCRT) is the conventional treatment for locally advanced rectal cancer (LARC). It improves R0 resection rate and reduces local recurrence rate, but it cannot improve long-term oncological outcomes. It also causes several radiotherapy-related side effects. In recent years, some studies have shown that neoadjuvant chemotherapy (NCT) may be noninferior to Lc-NCRT. Therefore, we systematically evaluated the efficacy and safety of NCT and Lc-NCRT for LARC.

**Methods:**

Cochrane Library, Embase, PubMed, WanFang Data, and CNKI were systematically searched as the relevant literature. The literature was screened independently by two groups, and data were extracted and evaluated for bias. A meta-analysis was performed using Revman5.4 software. The primary outcomes were tumor response to neoadjuvant therapy and long-term oncological outcomes.

**Results:**

A total of 17 studies with 5,168 cases (1,957 cases in NCT and 3,211 cases in Lc-NCRT) were included in our meta-analysis. Compared with the Lc-NCRT group, although the NCT group had a lower pCR rate [RR = 0.65, 95% CI (0.56–0.75), *P* < 0.0001], less downstaging [RR = 1.11, 95%CI(1.03–1.19), *P* = 0.06] and more adverse events of neoadjuvant therapy [RR = 1.11, 95% CI (1.03–1.19); *P* = 0.06], it had no difference in long-term survival outcome [3-year overall survival: HR = 1.13, 95% CI (0.70–1.83), *P* = 0.62; 3-year disease-free survival: HR = 1.16, 95% CI (0.96–1.39), *P* = 0.12; 3-year local recurrence-free survival: HR = 1.36, 95% CI (0.9–2.08), *P* = 0.15] and serious adverse events [RR = 0.84, 95% CI (0.45–1.57), *P* = 0.58] from the Lc-NCRT group. Moreover, the incidence of anastomotic leakage [RR = 0.48, 95% CI (0.34–0.45)] and permanent stoma rate [RR = 0.7, 95% CI (0.58–0.84), *P* < 0.0001] after operation was lower in the NCT group.

**Conclusion:**

NCT is a potential option for the treatment of LARC as it is beneficial for improving the sphincter preservation rate and reducing anastomotic leakage, the long-term oncological outcome is considerable, and the safety is controllable. Larger randomized controlled trials (RCT) with longer follow-up data are needed to clarify the specific regimens of NCT and the risk stratification of rectal cancer.

**Systematic Review Registration:**

https://www.crd.york.ac.uk/PROSPERO/home identifier, CRD42024579586.

## 1 Introduction

For nearly two decades, the standard treatment for LARC has been a model that included fluorouracil-based Lc-NCRT, total mesorectal excision (TME), and adjuvant chemotherapy (1). Compared with postoperative adjuvant therapy alone, Lc-NCRT has improved the quality of TME, increased the R0 resection rate, and reduced the local recurrence rate to less than 10% (2, 3), but it seems hard to improve the long-term survival outcome (2, 4). Although it can reduce the toxicity of chemoradiotherapy compared to postoperative adjuvant therapy, the uninterrupted course of Lc-NCRT for 28 days also makes it difficult for the patients to comply fully. Neoadjuvant radiotherapy causes pelvic tissue fibrosis, and autonomic nerve damage is associated with both short- and long-term morbidities. It may increase the difficulty of surgery and the incidence of postoperative anastomotic leakage, leading to a high stoma rate, and is a risk factor for low anterior resection syndrome. At the same time, it also affects fertility and sexual function and weakens pelvic bone marrow hematopoietic function (5–8). Moreover, the early use of systemic chemotherapy may reduce tumor micrometastases, and Lc-NCRT may delay the initiation of systemic therapy and provide a time window for the development of micrometastases, which may make distant recurrence a major cause of death in LARC (9). Therefore, to improve patient compliance, reduce the adverse events of neoadjuvant therapy, and improve long-term survival, some centers have explored the role of NCT in LARC, and it seems that NCT alone may result in an acceptable tumor response and disease-free survival (10–17), while others have yielded mixed results (18, 19). Many of these studies were small-sample, retrospective, and single-arm trials. Considering the inconsistent results of the published studies, we hope to compare the clinical efficacy of NAC and Lc-NCRT in the treatment of LARC through a systematic review and meta-analysis in order to obtain a higher level of evidence.

## 2 Methods

This study was performed according to the current Preferred Reporting Program for Systematic Reviews and Meta-analyses (PRISMA) (20) checklist and guidelines for methodological quality of systematic reviews AMSTER. It was registered at PROSPERO (registration number CRD42024579586).

### 21 Literature search strategy

Studies were searched by computer in public databases, including Cochrane Library, Embase, PubMed, WanFang Data, and CNKI. The search protocol used subject words in combination with free words to search the original studies of NCT compared to Lc-NCRT in the treatment of LARC, without limit in the search language. The search terms were “rectal neoplasm”, “chemotherapy”, “neoadjuvant”, and “chemoradiotherapy”, and the list of references of the retrieved studies was screened to identify citations that may be relevant to the analysis. The last literature search was conducted on September 9, 2024. Two researchers independently screened the retrieved literature and assessed the eligibility of each study included in the meta-analysis. Differences should be resolved through consensus and, if necessary, through meetings.

### 2.2 Inclusion and exclusion criteria of literature

The inclusion criteria were as follows: (1) biopsy specimen diagnosed as rectal adenocarcinoma, including mucinous adenocarcinoma and signet ring cell carcinoma, (2) staging at a locally advanced stage, TanyN+M0 and T3/4NanyM0 included, (3) intervention measures including NCT and Lc-NCRT, and (4) source data study with results including at least one of oncology, safety, and surgery-related indicators. The exclusion criteria were as follows: (1) rectal tumors of other pathological types, including stromal tumors and neuroendocrine tumors, (2) uncontrolled studies, (3) stage I or IV medical records, (4) data not derived from the original study, and (5) Newcastle–Ottawa Scale (NOS) score less than 6 for cohort studies. The most recent study was selected for inclusion if duplicate or overlapping articles were published by the same institution and researcher.

### 2.3 Data extraction

Two researchers independently extracted the literature data. When there were differences, they were verified by a third one, and the final analysis data were discussed and determined. The data we extracted were as follows:(1) the general data of the literature included the first author’s name, years of publication, region, study type, propensity score or not, number of study centers, enrollment time, and sample size enrolled in each group, (2) basic information of patients, including age, body mass index (BMI), sex, Eastern Cooperative Oncology Group (ECOG), tumor location, pathological type, clinical and pathological stage, American Society of Anesthesiologists (ASA) score, etc., (3) treatment regimen, neoadjuvant treatment regimen, cycle, postoperative adjuvant regimen, adverse events of adjuvant therapy, etc., (4) basic information of surgery, including operation type, sphincter preservation rate, stoma rate, R0 resection rate, pathological results, and postoperative complications, and (5) oncology outcomes included pathological complete response (pCR), downstaging, disease control rate (DCR), objective response rate (ORR), overall survival (OS), disease-free survival (DFS), local recurrence-free survival (LRFS), and distant metastasis-free survival (DMFS). The primary outcomes of this study were oncological outcomes, and the secondary outcomes were adverse events of chemoradiotherapy, R0 resection rate, sphincter preservation rate, and surgical complications. If only K-M plots are provided without HR data, HR is converted using the method of Jayne F. Tierney (21), and valid data that could not be extracted were not included in the meta-analysis. For studies with multiple chemoradiotherapy groups, only fluorouracil-based Lc-NCRT was included. For those with multiple NCT regimens, the results were combined for meta-analysis and separately included in the subgroup analysis. If the PSM study had similar data before and after matching, then the matched data were included in the meta-analysis.

### 2.4 Quality assessment

Two researchers independently evaluated the quality of the literature, and all disagreements were resolved by consensus. For RCT studies, the Cochrane Risk Bias Assessment Tool was implemented using the Revman 5.4 software. For cohort studies, the Newcastle–Ottawa Quality Assessment Tool (NOS scale) was used, which rates the quality of eight items in three domains: selectivity (up to four points), comparability (up to two points), and outcome (up to three points). The higher the score, the higher the quality is.

### 2.5 Statistical analysis

Meta-analysis was performed using Revman5.4 software. Relative risk (RR), mean difference (MD), hazard ratio (HR), and their corresponding 95% confidence intervals (95% CI) were calculated for dichotomous data and continuous and survival data, respectively. Q test and *I* ^2^ test analyses were used to examine heterogeneity in the literature. If heterogeneity was low (*I* ^2^ < 50%), the pooled estimate was calculated using the fixed-effects method. Otherwise, a random-effects model was used. For moderate to high heterogeneity, one-way sensitivity and subgroup analyses were used to explore the source of heterogeneity. Subgroup analysis according to study type (RCT or not, PSM or not), study publication time (before median publication time vs. later than median publication time), NCT regimens (FOLFOX vs. CAPOX, two-drug vs. three-drug, etc.), study center (multicenter vs. single-center), sample size (≥median sample size vs. <median sample size), and population characteristics (phase II vs. III, T4 vs. T1–3, N- vs. N+, EMVI+ vs. EMVI-, etc.). A funnel plot was used to test the stability of the meta-analysis for publication bias in studies that included more than 10 studies. A *p*-value of <0.05 for the combined data was significant.

## 3 Results

### 3.1 Study characteristics

**Figure 1:**
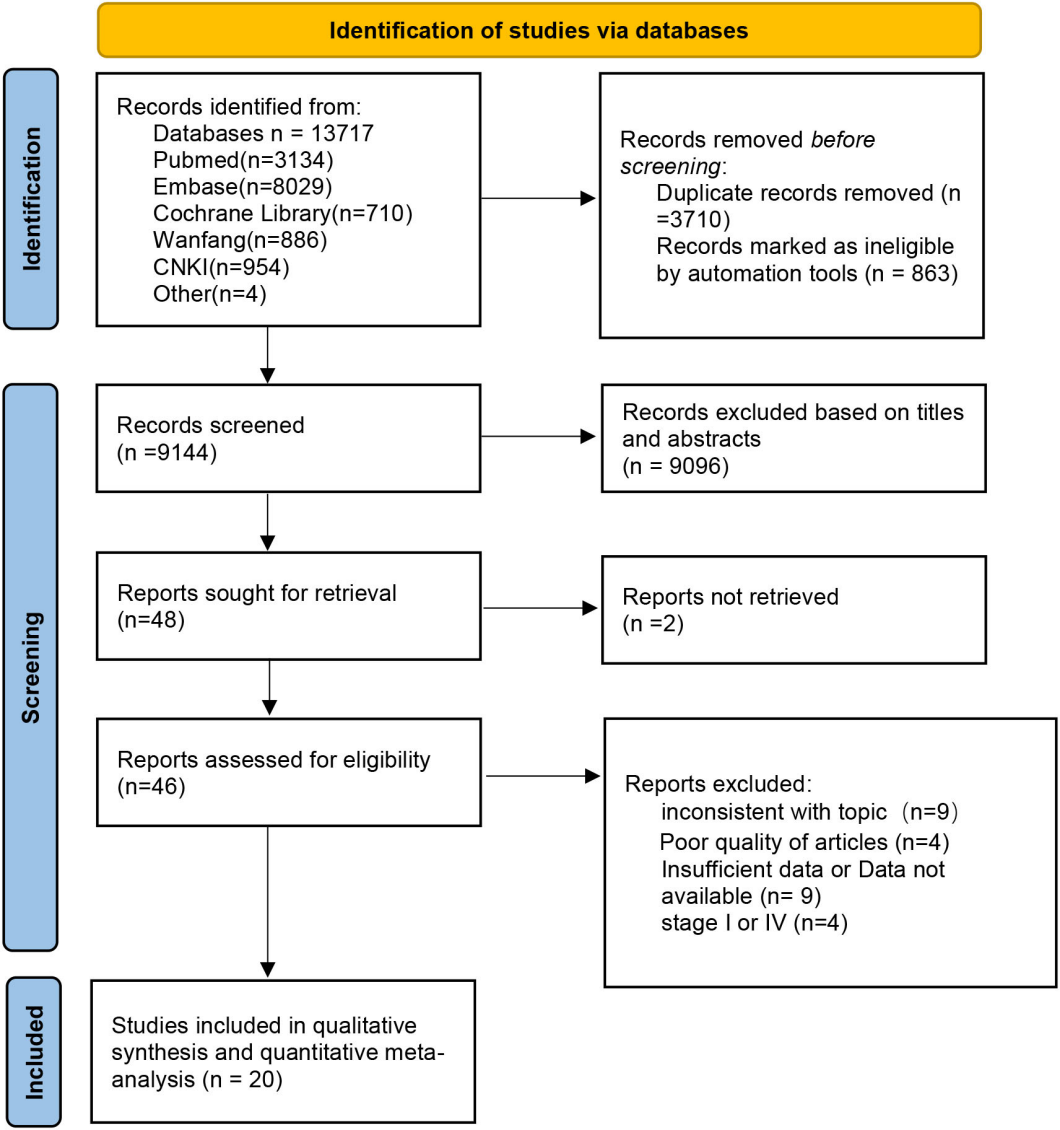
The PRISMA diagram for the selection of the studies.

A total of 13,717 articles were retrieved according to the initial search strategy, and 20 articles meeting the literature inclusion criteria were meta-analyzed. The literature screening process is illustrated in **Figure 1**. A total of 20 articles from 17 studies were included in this meta-analysis (three RCTs reported initial (13, 17, 22) and final results (15, 23, 24) in two separate studies). A total of 5,168 neoadjuvant patients were included in the analysis (NCT, 1,957 cases; Lc-NCRT, 3,211 cases), among which 5,106 cases received surgery (NCT, 1,926 cases; Lc-NCRT, 3,180 cases).

There were three RCTs (13, 15, 17, 22–24); the rest were cohort studies, five of which were propensity-score-matched (25–30). Only two studies were from the USA and Europe (12, 17), and the rest were from Asia. Seven were multi-center results (13, 15, 17, 28, 30, 31), nine studies have mid- or long-term oncology outcomes (14, 15, 23–27, 29, 30), and five studies were multi-arm studies (including surgery alone, short-course radiotherapy group, chemotherapy with different regimens, etc.) (12, 13, 25, 31, 32), only long-course cases of radiotherapy concurrent with fluorouracil-based chemotherapy were included as controls.

As shown in **Table 1**, CAPOX and FOLFOX (including mFOLFOX6 and FOLFOX4) were the most common NCT regimens, and SOX (14) and FOLFIRINOX (17) were included in some earlier studies. In cohort studies, all patients underwent surgery, and patients from Japan underwent selective lateral lymph node dissection(LLND) (14, 35), while RCT studies had slightly fewer surgical samples than neoadjuvant therapy samples. Most of the studies performed postoperative chemotherapy, but only seven provided data (13, 14, 22, 25, 26, 28, 30). There seems to be no significant difference in preoperative T stage, N stage, clinical stage, age, and gender between groups. Some studies also performed subgroup analysis according to distance from the anus (24, 27) and clinical stage (30), and the patients were all EMVI+ of LARC in one study (25). One study adopted different neoadjuvant regimens according to the stratum. This meta-analysis extracted data from the stratum of favorable response for the investigator and only compared NCT and Lc-NCRT in this stratification (17). The characteristics of the study, including population, are presented in **Table 1**.

**Table 1:** Group assignment and population characteristics of each study.

Author	Groups	Sample size			Treatment regimens (neoadjuvant treatment + surgery + adjuvant treatment)	Agemean (SD)/median (range)	Male/female	cT1/2/3/4	cN0/1/2	cII/III
		Neoadjuvant	Surgery	Adjuvant						
Qi Sun 2024 (33)	NAC	67	67		mFOLFOX6/CAPOX + TME + NA	56 (49–62.5)	24/43	0/0/41/26		20/47
	Lc-NCRT	51	51		mFOLFOX6/CAPOX and 45–50.4 Gy of RT + TME + NA	58 (49–65)	22/29	0/1/34/16		21/30
Yu Shen 2024 (34)	NAC	61	61		At least 4 cycles of CAPOX + TME + NA	58.54 (9.86)	47/14	0/0/53/8	23/37/1	
	Lc-NCRT	116	116		50.4 Gy of RT with concurrent 5-FU or CAP + TME + NA	55.24 (10.10)	76/40	0/0/71/45	8/47/61	
Jingjing Wu 2023 (27)	NAC	104	104	46	mFOLFOX6 or 5-FU + TME + 4 cycles of mFOLFOX6 or 5-FU	53.6 (12.9)	74/30	0/1/92/11	23/47/34	23/81
	Lc-NCRT	104	104	67	5 cycles of de Gramont or mFOLFOX6 and 46–50.4 Gy of RT + TME + 7 cycles of de Gramont or mFOLFOX6	52.9 (11.4)	73/31	0/4/87/13	23/44/37	23/81
Yimin Han 2023 (26)	NAC	54	54		4 (range, 2–6) cycles of CAPOX or FOLFOX + TME + 3 (range, 1–6) cycles of CAPOX or FOLFOX	60 (34–75)	41/13	0/0/35/19	7/20/27	7/47
	Lc-NCRT	101	101		2 (range, 1–8) cycles of (CAPEOX or FOLFOX) and 50 Gy of RT concurrent with CAP and 2 (range, 1–8) cycles of (CAPEOX or FOLFOX) + TME + 4 (range, 2–7) cycles of adjuvant chemotherapy	68 (31–75)	73/28	0/0/40/61	7/27/67	7/94
Weijing Mei 2023 (22), Peirong Ding 2023 (15)	NAC	291	272	235	4 cycles of CAPOX + TME + 4 cycles of CAPOX	60 (31–75)	188/112	0/16/201/83	92/147/61	92/208
	Lc-NCRT	284	261	222	45–50 Gy of RT concurrent with oral CAP + TME + 6 cycles of CAPOX	60 (28–75)	177/112	0/11/202/76	77/133/79	77/212
Mo Chen 2023 (25)	NAC	238	238		NA	>60 year(53.4%)	178/60	0/1/142/95	10/84/144	9/229
	Lc-NCRT	80	80		NA	>60 year(61.2%)	59/21	0/0/47/33	4/27/49	3/77
Hongxia Yan 2022 (16)	NAC	52	52	46	3 cycles of mFOLFOX4 + TME + more than 4 cycles of AC	>60 year (59.6%)	35/17	0/0/20/32		50/2
	Lc-NCRT	76	76	67	40–50 Gy of RT concurrent with 3 cycles of mFOLFOX4 + TME + more than 4 cycles of AC	>60 year (67.1%)	50/26	0/0/32/44		75/1
Xuan Zhao 2022 (29)	NAC	92	92		6–8 cycles of mFOLFOX6 or CAPOX + TME + AC for stage pIII or high risk of stage pII	61.23 (8.64)	74/18	0/3/48/41	6/34/52	6/86
	Lc-NCRT	92	92		3–4 cycles of mFOLFOX6 or CAPOX + 50 Gy of RT concurrent with 5-FU + 1 cycle of mFOLFOX6 or CAPOX + TME + AC	60.60 (8.61)	70/22	2/3/38/49	4/38/50	4/88
Wei Jiang 2022 (28)	NAC	100	100		4–6 cycles of mFOLFOX6 + TME + mFOLFOX6	>65 year (25%)	65/35			38/62
	Lc-NCRT	107	107		5 cycles of mFOLFOX6 and 46–50.4 Gy of RT + TME + mFOLFOX6	>65 year (15.9%)	72/35			62/45
Philippe Rouanet 2022 (23), 2017 (17)	NAC	11	10		4 cycles of FOLFIRINOX + TME + 6 cycles of FOLFOX for ypT2–4 or ypN +	66.0 (44–78)	5/6	All are T3		2/9
	Lc-NCRT	19	19		4 cycles of FOLFIRINOX + standard Cap 50 CRT + TME + 6 cycles of FOLFOX for ypT2–4 or ypN +	63.0 (39–75)	11/8	All are T3		4/14 and 1missing
Gongqin Chen 2021 (32)	NAC	46	46		3–4 cycles of CAPOX + TME + NA	>60 year(54.3%)	27/19	0/0/38/8	0/10/32	0/42
	Lc-NCRT	42	42		50 Gy in 25 fractions of RT concurrent with oral CAP + CAPOX + TME + NA	>60 year(26.2%)	29/13	0/0/31/11	0/10/32	0/46
Fang He 2020 (30)	NAC	565	565	485	FOLFOX or CAPOX + TME + AC (determined by MDT)		388/177	1/15/433/122	147/418/0	147/418
	Lc-NCRT	1852	1852	1545	50.0 Gy of RT concurrent with 5-FU + TME + AC (determined by MDT)		1255/597	7/97/1660/898	428/1421/3	428/1361
Yingbin Wang 2020 (31)	NAC	58	58		3 cycles of CAPOX + TME + CAPOX or second-line chemotherapy regimens					
	Lc-NCRT	58	58		40–45 Gy of RT with concomitant CAPOX + TME + 6–8 cycles of CAPOX					
Yanhong Deng 2019 (24), 2016 (13)	NAC	163	152	141	4–6 cycles of mFOLFOX6 + TME + 6–8 cycles of mFOLFOX6	54.1 (12.1)	108/57	0/1/114/50	46/76/43	46/119
	Lc-NCRT	157	149	134	5 cycles of de Gramont AND 46–50.4 Gy of RT + TME + 7 cycles of de Gramont	54.0 (11.9)	103/62	0/8/100/57	30/88/47	37/128
Kentaro Sato 2019 (35)	NAC	16	16		3 courses of SOX + TME with or without of LLND + NA	67.5 (43–77)	14/2			
	Lc-NCRT	10	10		40–45 Gy of RT with concomitant 5-FU + TME with or without of LLND + NA	66 (53–71)	5/5			
Felipe Quezada-Diaz 2018 (12)	NAC	12	12		8 cycles of mFOLFOX6 or 5 cycles of CAPOX or FLOX + TME + NA	47.5 (25–77)	6/6			
	Lc-NCRT	34	34		50 or 50.4 Gy in 25 or 28 fractions of RT with concurrent infusional FU or oral CAP + TME + NA	54.0 (34–78)	21/13			
Takashi Okuyama 2018 (14)	NAC	27	27	9	For wild-type KRAS:2 cycles of SOX + cetuximab + TME + 5-FU-leucovorin or capecitabine or SOX or FOLFOX for yPT4/ypN +/ypCRM +. For non-wild-type KRAS:1–2 cycles of SOX or 2–9 cycles of mFOLFOX6 or 2–3 cycles of XELOX + TME + 5-FU-leucovorin or capecitabine or SOX or FOLFOX for patients with yPT4/ypN +/ypCRM+	66.0 (40–79)	17/10	0/0/24/3	0/18/9	0/27
	Lc-NCRT	28	28	12	45 Gy of RT with concomitant of 5-FU + TME + 5-FU-leucovorin or capecitabine or SOX or FOLFOX for patients with yPT4/ypN+/ypCRM+	68.0 (42–78)	16/12	0/0/22/5	0/17/11	0/28

de Gramont: leucovorin 400 mg/m^2^ intravenous drip, then fluorouracil 400 mg/m^2^ intravenous drip, fluorouracil 2.4 g/m^2^ by 48-h continuous intravenous infusion; Cap 50 = 50 Gy irradiation with concomitant 1,600 mg/m^2^/day capecitabine; FOLFIRINOX:4 cycles of 180 mg/m^2^ irinotecan, 85 mg/m^2^ oxaliplatin, 200 mg/m^2^ elvorin, and 5-fluorouracil (400 mg/m^2^ bolus followed by 2,400 mg/m^2^ continuous infusion for 46 h) delivered over 8 weeks (day 1 = day 15).

### 3.2 Quality evaluation of the included literature

**Figure 2:**
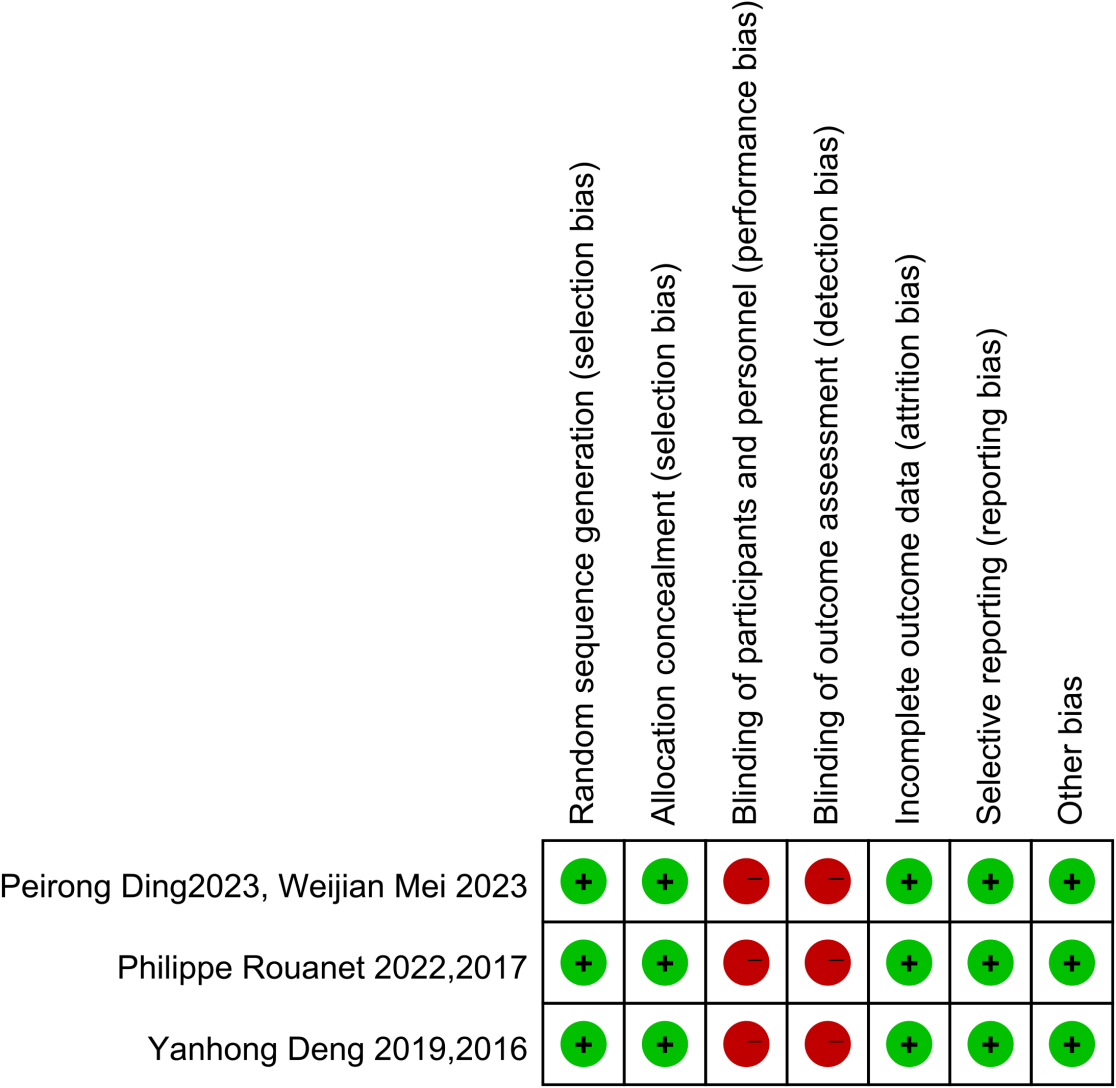
Risk of bias summary for three randomized controlled trials.

Of the 20 included studies, 14 were cohort studies. The quality of the literature was using the NOS scale from eight points in three aspects of selectivity, control line, and result. The scores in the literature are listed in **Table 2**. The higher the score is, the higher the quality is, and the NOS scores of all studies are more than 5. The Cochrane bias risk assessment tool was used to assess the article quality of the three RCTs (**Figure 2**), and there was no significant bias except for the blinding method. Therefore, the overall quality of the 20 articles from these 17 studies was considered to be good.

**Table 2:** General characteristics of each study and NOS scores of cohort studies.

First author	Year	Case enrollment period	Origin	Study type	Number of centers	PSM	NOS	Patients’ inclusion criteria			
								Age(year)	Distance from anus (cm)	Stage	Others
Qi Sun (33)	2024	January 2019 to 2021 December	China	Cohort	1	N	7		≤12	T3-4/N1-2	Adenocarcinoma, R0 resection
Yu Shen (34)	2024	March 2016 to December 2019	China	Cohort	1	N	6	18–75	≤12	II/III	MRI before and after neoadjuvant treatments, TME surgery and complete pathological report
Jingjing Wu (27)	2023	February 2012 to April 2015	China	Cohort	1	Y	9	20–74		II/III	ASA = 1–3, rectal adenocarcinoma, R0 resection
Yimin Han (26)	2023	January 2016 to June 2021	China	Cohort	1	Y	8			II/III	Received TME
WeiJian Mei (22) Peirong Ding (15)	2023 2023	June 2014 to October 2020	China	RCT phase III CONVERT	21			18–75	≤12	cT2N+/cT3-4aNany	Pathologically confirmed, no previous treatment, ECOG ≤ 1, adequate hematologic, liver, and renal function
Mo Chen (25)	2023	January 2013 to October 2021	China	Cohort	NA	Y	9			LARC, mrEMVI+	Adenocarcinoma, no distant metastases, curative treatment, and TME
Xuan Zhao (29)	2022	June 2016 to October 2021	China	Cohort	1	Y	8	18–80	≤12	LARC	Adenocarcinoma, R0 resection
Hongxia Yan (16)	2022		China	Cohort	1	N	7		≤10	T4 or T3-4N+	W.H.O. PS = 0–2, surgery performed by the same treatment group
Wei Jiang (28)	2022	January 1, 2018 to August 31, 2020	China	Cohort	2*	N	6	18–75	≤12	II/III	Histologically confirmed, radical anterior rectal resection
Philippe Rouanet (17, 23)	20222017	May 2011 and October 201	France	RCT phase II GRECCAR4	16			≥18	≥1	LARC mriT3≥c or mriT4, CRM ≤ 1 mm	ECOG ≤2, adequate hematological, hepatic, and renal function
Gongqin Chen (32)	2021	April 2018 to March 2020	China	Cohort	1	N	7	18–80	≤12	cT3-4N+	Histologically confirmed, ECOG ≤ 2; laparoscopic surgery is feasible, The estimated survival time was >12 months
Fang He (30)	2020	January 2010 to December 2018	China	Cohort	Multicenter	Y	8		≤15	II/III	
Yingbin Wang (31)	2020	January 2013 to January 2018	China	Cohort	2		7		≥3	cT3–4 N0-2M0	Adenocarcinoma, ECOG < 2
Yanhong Deng (13, 24)	2019 2016	June 9, 2010 to February 15, 2015	China	RCT phase III FOWARC	15			18–75	≤12	II/III	Adenocarcinoma, suitable for curative resection, ECOG = 0/1, adequate hematologic, liver, and renal function
Kentaro Sato (35)	2019	NAC (October 2015 to June 2016) Lc-NCRT (June 2002 to January 2012)	Japan	Cohort	1	N	6			LARC	
Felipe Quezada-Diaz (12)	2018	November 1, 2011 to August 31, 2017.	USA	Cohort	1	N	7		≤10	I (for TNT group) II/III	Adenocarcinoma, sphincter-preserving TME
Takashi Okuyama (14)	2018	April 2010 and February 2016	Japan	Cohort	1	N	8	<80		cT3–4 N+M0	Previously untreated, requiring surgery, W.H.O. PS = 0/1, histologically confirmed

PSM, propensity score match; NOS, Newcastle-Ottawa Scale; TME, total mesorectal excision; LARC, locally advanced rectal cancer; CRM, circumferential resection margin; ECOG, Eastern Cooperative Oncology Group; W.H.O. PS, World Health Organization Performance Status; ASA, American Society of Anesthesiologists.

### 3.3 Meta-analysis results

We systematically evaluated the effects of neoadjuvant therapy on LARC in terms of tumor response to neoadjuvant therapy, short- and long-term survival outcomes, safety of neoadjuvant therapy, surgical complications, and anal retention.

#### 3.3.1 Tumor response

**Figure 3:**
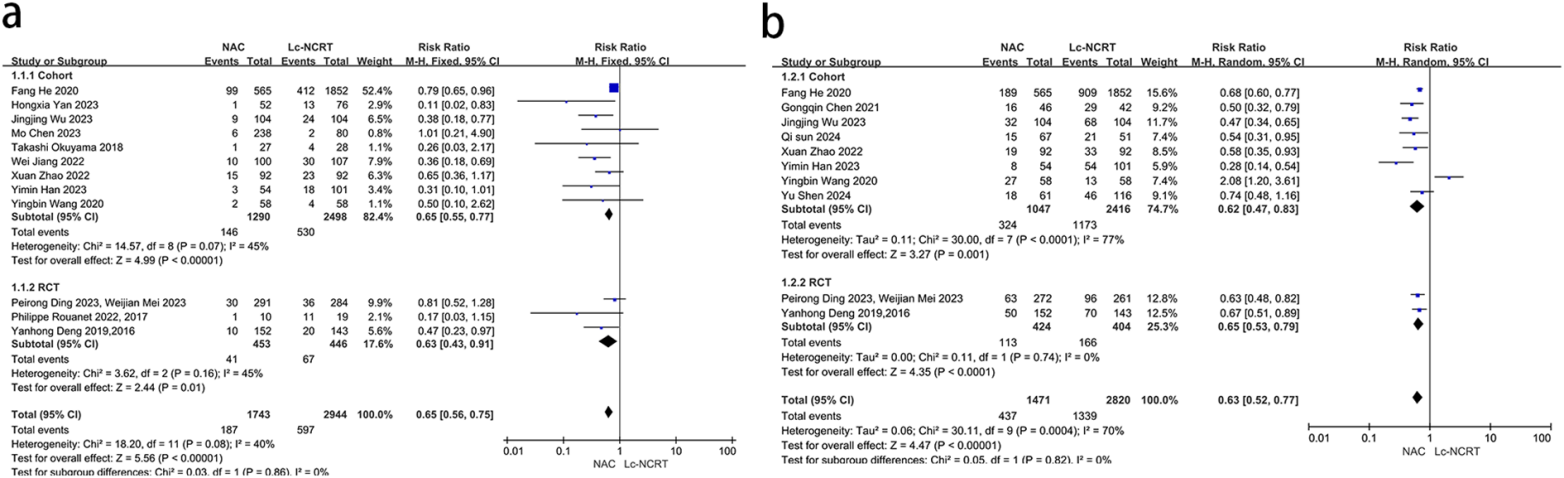
Forest plots of **(a)** pCR and **(b)** pTRG0–1 between groups of NCT and Lc-NCRT.

pCR results were reported in 12 studies (**Figure 3a**). There was mild heterogeneity among the studies (*I* ^2^ = 40%, chi-square test, *P* = 0.08). A fixed-effect model was used. The results showed that the probability of achieving pCR in the NCT group was less than that in the Lc-NCRT group, and the difference was statistically significant [RR = 0.65, 95% CI (0.56–0.75), *P* < 0.0001]. The results of the subgroup analyses were consistent.

The results of pTRG0–1 were reported in 10 studies (**Figure 3b**). There was moderate heterogeneity among the studies (*I* ^2^ = 70%, chi-square test, *P* = 0.0004). A random-effect model was used. The results showed that the probability of achieving pTRG0–1 in the NCT group was less than that in the Lc-NCRT group, and the difference was statistically significant [RR = 0.63, 95% CI (0.52–0.77), *P* < 0.0001]. There was no heterogeneity in the RCT subgroup, while there was still high heterogeneity in the cohort subgroup. However, the results did not change.

**Figure 4:**
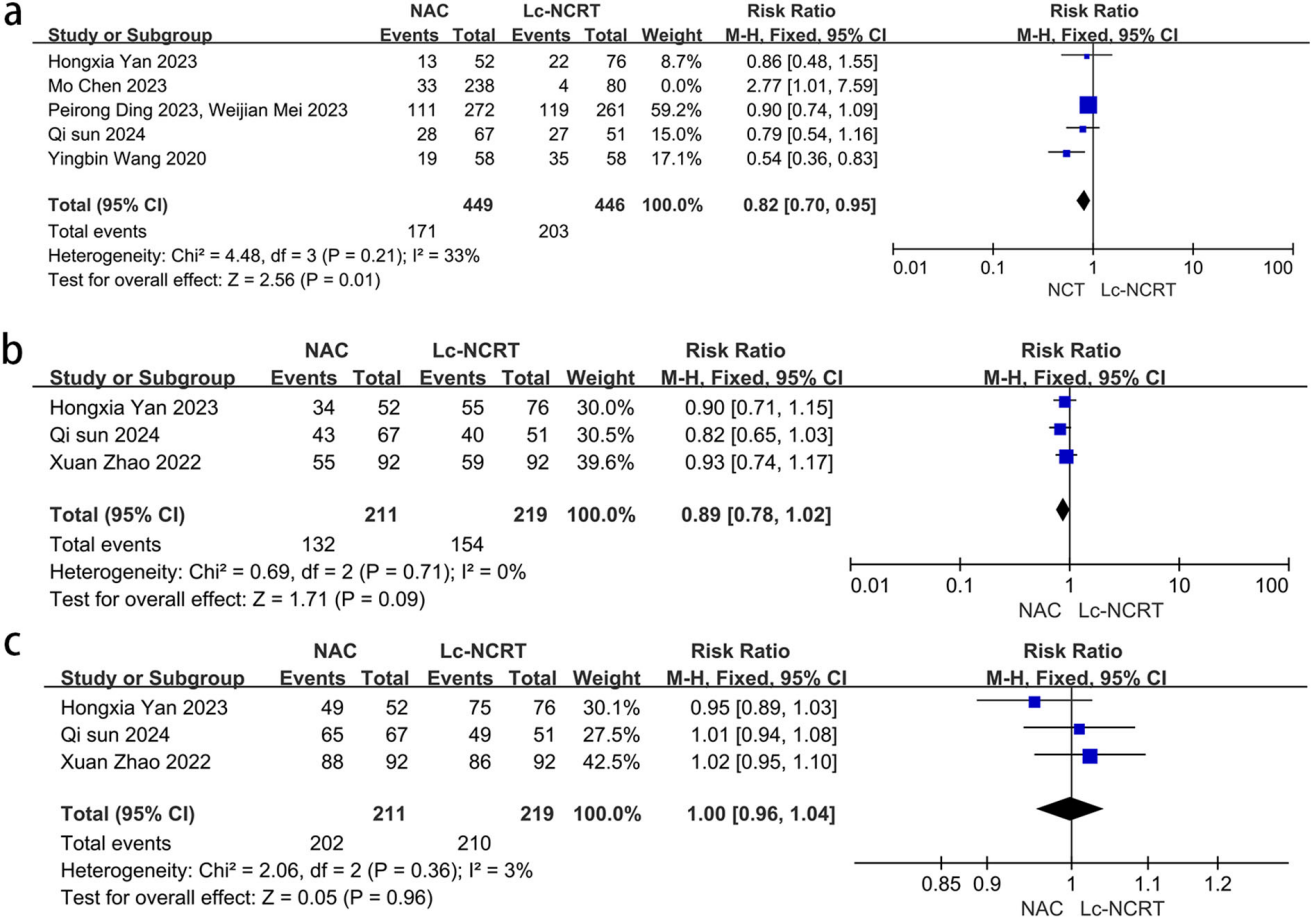
|Forest plots of **(a)** downstaging and **(b)** ORR. **(c)** DCR between groups of NCT and Lc-NCRT.

Five studies reported the outcome of downstaging (**Figure 4a**), and there was moderate heterogeneity among the studies (*I* ^2^ = 61%, chi-square test, *P* = 0.61). The sensitivity analysis suggested that the study by Mo Chen (11) may be the source of heterogeneity (*I* ^2^ = 33%, chi-square test, *P* = 0.21). Considering that the cases in this study were all EMVI-positive, which was different from other studies, they were excluded. A fixed-effect model was adopted, and the results showed that the downstaging rate in the NCT group was lower than that in the Lc-NCRT group [RR = 0.82, 95% CI (0.70–0.95); *P* = 0.01].

Three studies reported the outcomes of ORR and DCR (**Figures 4b, c**). There was no heterogeneity among these studies (ORR: *I* ^2^ = 0%, chi-square test, *P* = 0.71 and DCR: *I* ^2^ = 3%, chi-square test, *P* = 0.36). The results showed that there was no significant difference in direct ORR and DCR between the NCT and the Lc-NCRT groups using a fixed-effects model [ORR: RR = 0.89, 95% CI (0.78–1.02), *P* = 0.09; DCR: RR = 1.0, 95% CI (0.96–1.04), *P* = 0.96].

#### 3.3.4 Survival outcomes

**Figure 5:**
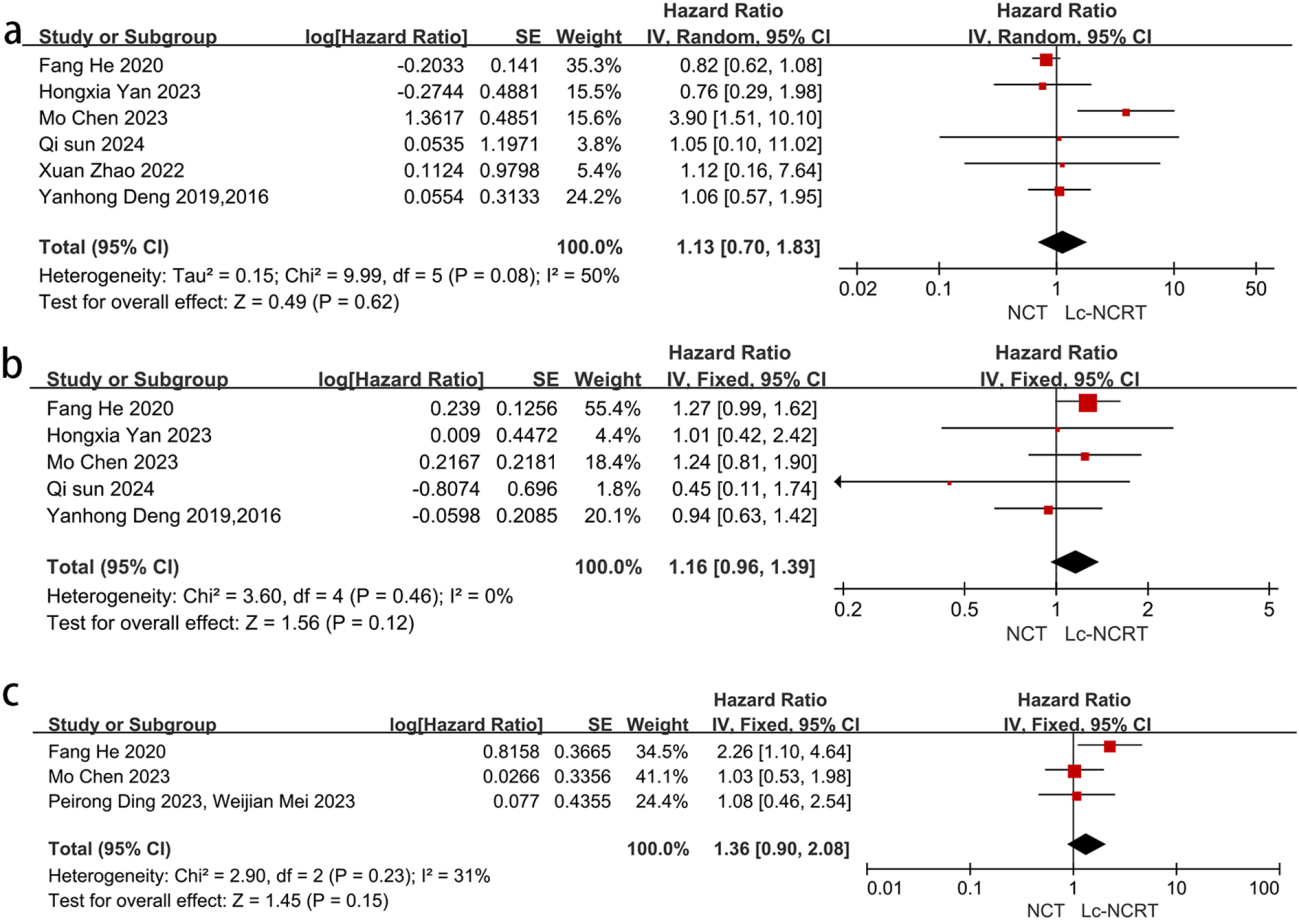
Forest plots of **(a)** 3-year OS and **(b)** 3-year DFS. **(c)** 3-year LRFS between groups of NCT and Lc-NCRT.

Nine papers reported long-term oncological outcomes, including 3 years of OS, DFS, and LRFS (**Figure 5**). Only one or two studies reported survival outcomes at 2 and 5 years, respectively; therefore, no meta-analysis was performed.

Six studies reported a 3-year OS. The meta-analysis showed moderate heterogeneity among the studies (*I* ^2^ = 50%, chi-square test, *P* = 0.08). No significant difference in 3-year OS was observed between the two groups using a random-effects model [HR = 1.13, 95% CI (0.70–1.83), *P* = 0.62]. In the sensitivity analysis, Mo Chen (11) was considered as a heterogeneous source because all of the patients included in Mo Chen (11) were EMVI-positive. Heterogeneousness was reduced after the exclusion of Mo Chen’s study (*I* ^2^ = 0%, chi-square test, *P* = 0.95), and there was still no significant difference between the two groups using the fixed-effects model [HR = 0.85, 95% CI (0.67–1.08), *P* = 0.19].

Five studies reported 3-year DFS, and the meta-analysis showed no significant difference between the two groups in 3-year DFS [HR = 1.16, 95% CI (0.96–1.39), *P* = 0.12]. There was no heterogeneity among the studies (*I* ^2^ = 0%, chi-square test, *P* = 0.46).

Only three studies reported 3-year LRFS. There was mild heterogeneity among these studies (*I* ^2^ = 31%, chi-square *P* = 0.23), and the meta-analysis showed no significant difference between the two groups [HR = 1.36, 95% CI (0.90–2.08), *P* = 0.15].

#### 3.3.3 Adverse events of neoadjuvant therapy

**Figure 6:**
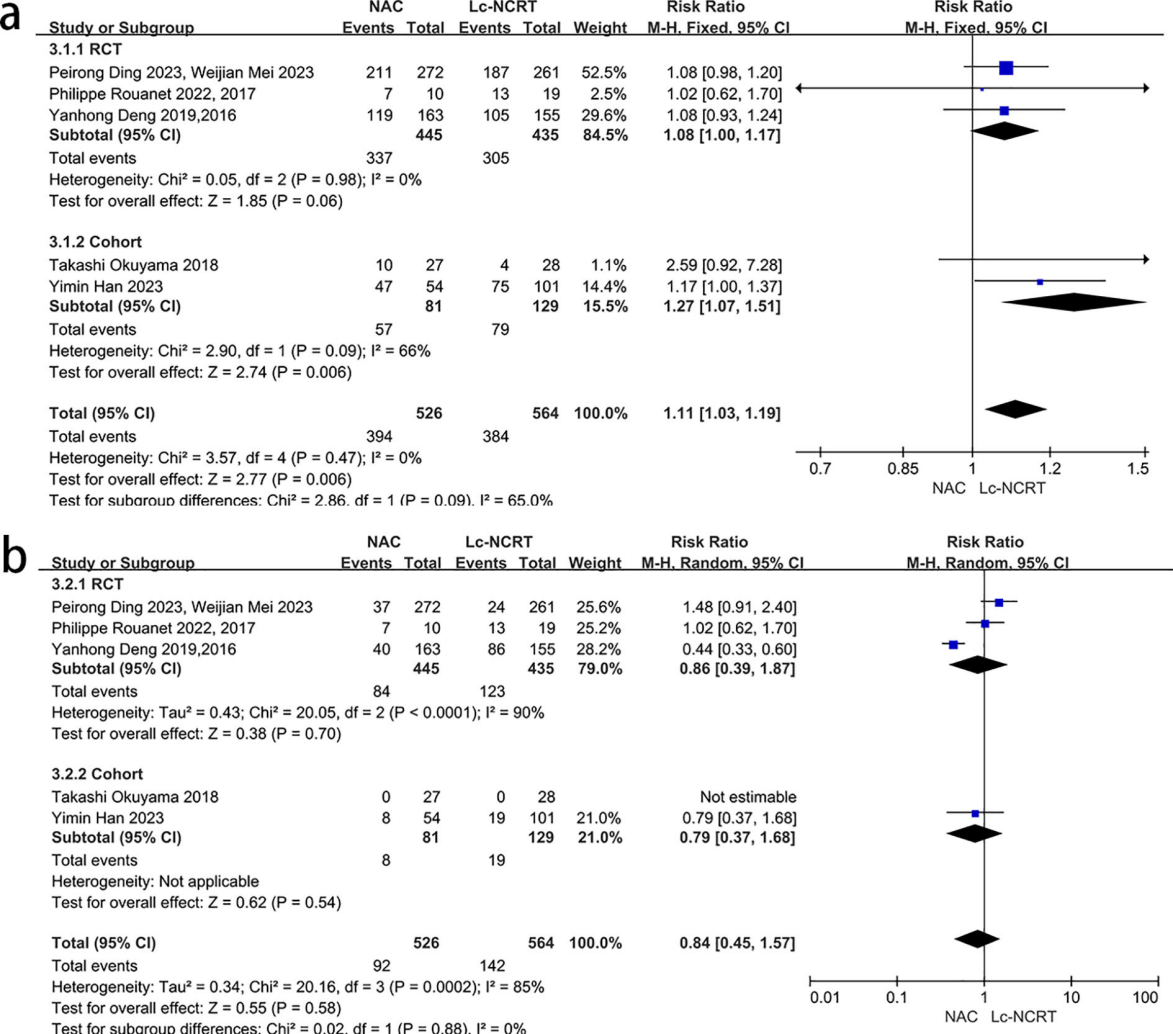
Forest plots of **(a)** adverse events and **(b)** severe adverse events of neoadjuvant therapy between groups of NCT and Lc-NCRT.

There was no heterogeneity among all of the five studies that reported adverse events of neoadjuvant therapy (*I* ^2^ = 0%, chi-square test, *P* = 0.47), and a fixed-effect model was used (**Figure 6a**). The results showed that there were more adverse events in the NCT group, with a statistically significant difference [RR = 1.11, 95% CI (1.03–1.19), *P* = 0.06]. However, the subgroup analysis suggested that there may be no significant difference between the two groups in the RCT subgroup [RR = 1.08, 95% CI (1.00–1.17), *P* = 0.006].

Five studies reported severe adverse events of neoadjuvant therapy, and there was high heterogeneity among the studies (*I* ^2^ = 85%, chi-square test, *P* = 0.0002) (**Figure 6b**). The subgroup analysis failed to identify the source of heterogeneity. The sensitivity analysis suggested that Yanhong Deng (2, 7) may be the source of heterogeneity, but there was no definite clinical indication to exclude it. Therefore, the random-effects model was used, and the results showed that there was no statistically significant difference between the two groups [RR = 0.84, 95% CI (0.45–1.57), *P* = 0.58]. The subgroup analysis results did not change.

#### 3.3.4 Surgical complications and anal retention

**Figure 7:**
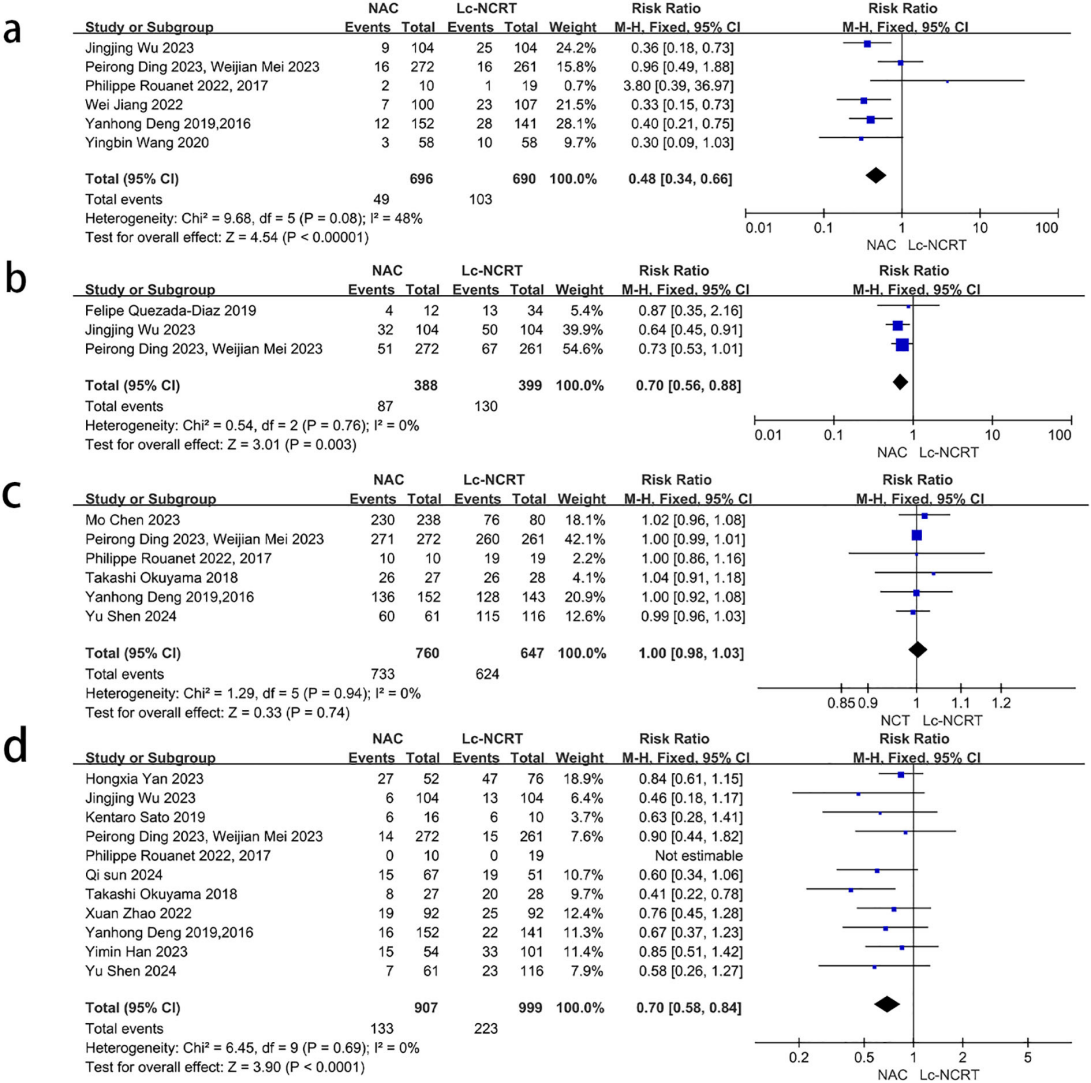
Forest plots of **(a)** R0 rate, **(b)** postoperative complications, **(c)** postoperative anastomotic leakage, and **(d)** permanent stoma rate between groups of NCT and Lc-NCRT.

Six articles reported R0 resection rates (**Figure 7a**). The meta-analysis showed that there was no significant heterogeneity among the studies (*I* ^2^ = 0%, chi-square test, *P* = 0.94), and no significant difference was found between the two groups [RR = 1.0, 95% CI (0.98–1.03), *P* = 0.74].

Three articles reported the total number of postoperative complications (**Figure 7b**), and there was no significant heterogeneity among studies (*I* ^2^ = 0%, chi-square test, *P* = 0.76). The postoperative complications in the NCT group were less than that in the Lc-NCRT group [RR = 0.70, 95% CI (0.56–0.88), *P* = 0.003]. Only Jingjing Wu reported that there were more grade III–IV complications in the Lc-NCRT group than in the NCT group. The incidence of postoperative anastomotic leakage was significantly lower in the NCT group (RR = 0.48, 95% CI (0.34–0.66), *P* < 0.00001), and there was slight heterogeneity (*I* ^2^ = 48%, chi-square test, *P* = 0.08) (**Figure 7c**). The sensitivity analyses suggested that Wei-Jian Mei (4) may be a source of heterogeneity, but there is no clinical evidence for its exclusion, and even its exclusion does not change the result.

A meta-analysis of 11 studies showed that the permanent stoma rate in the NCT group was significantly lower than that in the Lc-NCRT group [RR = 0.7, 95% CI (0.58–0.84), *P* < 0.0001], and there was no heterogeneity among these studies (*I* ^2^ = 0%, chi-square test, *P* = 0.69) (**Figure 7d**).

### 3.4 Bias analysis

**Figure 8:**
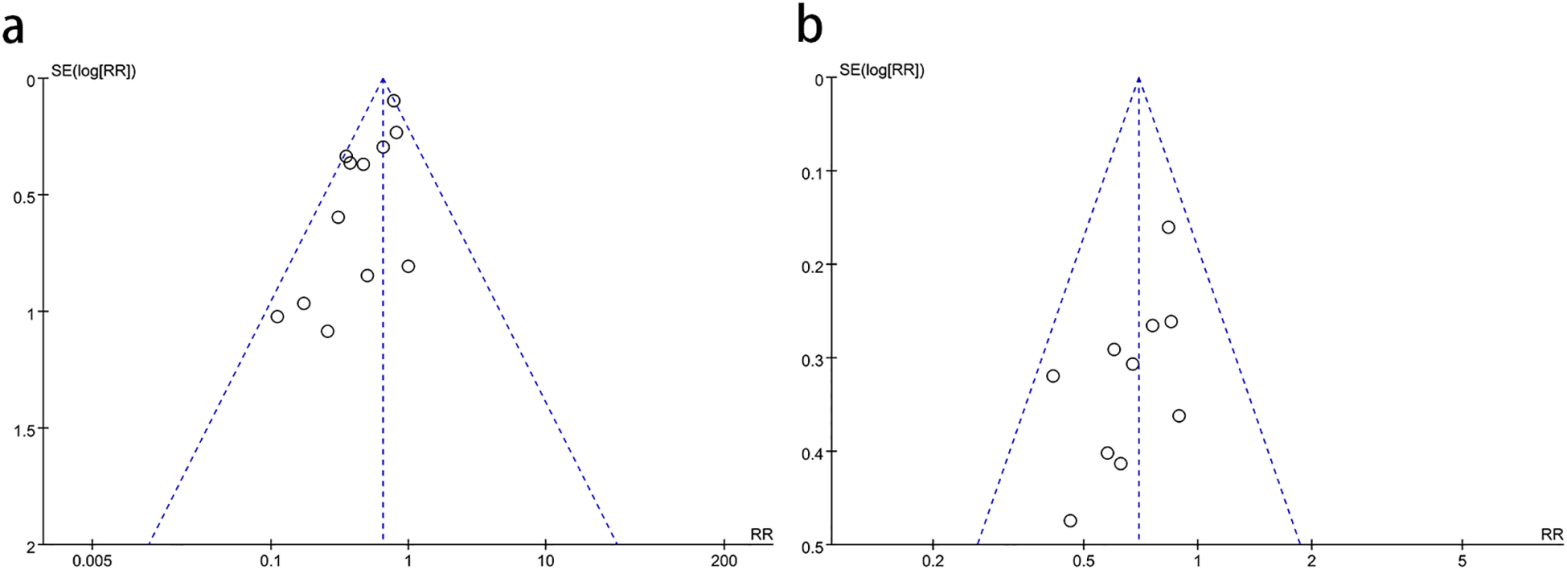
Funnel plots of **(a)** pCR and **(b)** permanent stoma rate.

We performed funnel plot analysis for the meta-analyses of more than 10 studies, and it can be seen that the scatter points fall within the funnel plot. However, the scatter points are not very symmetrical, indicating that there may be some publication bias (**Figure 8**).

## 4 Discussion

TME surgical techniques significantly reduce the risk of postoperative local recurrences (36). The primary purpose of preoperative chemoradiotherapy is tumor downstaging to obtain better R0 resection and TME quality in LRAC. However, in the past decade, laparoscopic and robotic technologies have developed rapidly, enabling surgeons to perform TME better (37–39). Various systemic treatment methods such as immunotherapy have also made great progress (40, 41). Therefore, overemphasis on high-intensity neoadjuvant radiotherapy to achieve pCR may increase adverse events, delay the timing of systemic therapy, and lead to distant metastasis (42). These therapeutic advances and conceptual changes pose a challenge to the Lc-NCRT model. Whether NCT alone can replace Lc-NCRT to create the same surgical conditions, achieve similar oncological outcomes, and avoid the side effects of radiotherapy has become a research hotspot.

Our meta-analysis comprehensively evaluated the differences between NCT and Lc-NCRT groups from four aspects—tumor response, long-term oncological outcomes, safety, and surgical-related indicators—and is the one with the highest quality of literature, the most reports of medium- and long-term oncology outcomes, and the most detailed subgroup analysis. Two similar meta-analyses were published in 2021 (43, 44), focusing on differences in short-term outcomes, and the conclusion was partly consistent with our study. However, the included literature was highly variable. Some of their studies included patients with stage I and IV disease (45–48), and some used surgery alone or other chemotherapy regimens as controls (47–49). When conducting a meta-analysis of anastomotic leak versus sphincter preservation, they repeatedly included data from the FOWARC study (5, 8, 13), which may lead to a bias. Our results showed that, although the pCR rate of NCT was lower than that of Lc-NCRT and the total adverse event rate of NCT was higher, there was no statistical difference in R0 resection rate and mid- and long-term oncological outcomes and neoadjuvant treatment toxicity above grade III. The incidence of surgical complications such as anastomotic leakage and the rate of permanent stoma were lower in the NCT than that in the Lc-NCRT group. There is still some controversy in short-course and non-FU-based chemoradiotherapy (50–52). Therefore, we included only fluorouracil-based neoadjuvant chemoradiotherapy as a control group.

In terms of tumor response, although there was no significant difference between the two groups in terms of DRR and ORR, postoperative pathological results such as pCR, TRG0-1, and pathological downstaging showed that NCT was inferior to Lc-NCRT, which differ from previous studies (43, 44). This difference may be due to the difference in sample sizes or because the imaging diagnosis could not fully reflect the pathological results and even for the heterogeneity among the cases in each study. As we can see, the meta-analysis results of tumor downstaging changed after excluding Mo Chen’s study (25). It may be because all of the patients included in the study were EMVI-positive, which was different from that of others. Although the patients in the NCT group had less pathological downgrading than those in the Lc-NCRT group, the pathological response does not reflect long-course survival outcomes. In this study, we found no difference between the two groups in 3-year OS, DFS, and LRFS. Unfortunately, only two articles involved distant metastasis-free survival (25, 30) and 5-year survival outcome (23, 27). Thus, it is difficult to perform a meta-analysis. Therefore, the effect of the NCT scheme on long-term survival of LRAC should be considered cautiously. Some conference abstracts indicated that the use of Lc-NCRT was independently associated with better OS than NCT alone (18). However, such database studies with non-original data did not meet the inclusion criteria of our study.

Regarding safety, we found more adverse events of neoadjuvant therapy in the NCT group than in the Lc-NCRT group, which may be related to the generally longer duration of chemotherapy in the NCT group but was balanced between the two groups in the RCT subgroups. These adverse events of neoadjuvant therapy were mainly of grades I–II, for there was no significant difference between the two groups in events greater than grade III. Overall, although more neoadjuvant adverse events occurred in the NCT group, they were manageable, and the safety was completely acceptable. Although the tumor response in the NCT group was inferior to that of Lc-NCRT, it did not reduce the R0 resection rate in our meta-analysis. On the contrary, the rates of permanent stoma, anastomotic leak, and surgical complications were significantly lower. Tissue damage caused by radiotherapy significantly increases the difficulty of the operation and thus affects the quality of the anastomosis (53). In addition, given the high incidence of anastomotic leakage and severe impairment of defecation control after radiotherapy, surgeons may prefer stoma, at least protective stoma, especially for patients with low rectal cancer (54).

There are obvious limitations in our studies. First, the included studies were mainly from Asia, with only two studies from Europe and America. Second, there were only three RCT studies, which was less than half of the total number of studies. Third, although patients of stage II–III were strictly included, the enrollment criteria of these studies are still slightly different. Fourth, the regimens and cycles of NCT are not completely unified. The chemotherapy regimens in the three RCTS were different. Fifth, the literature included in the analyses of long-term oncologic outcomes remains modest. Sixth, the scatter points in the funnel plot of this study were not very symmetrical, which may suggest the existence of publication bias. There may be multiple sources of bias. First, some articles with negative results may not have been published. Second, the database that we searched may not have covered all relevant literature, and no literature other than English and Chinese was retrieved. Third, the regimen and sequence of chemotherapy were inconsistent, and some studies of consolidation chemotherapy after radiotherapy may have resulted in favorable results for the radiotherapy group (9, 16, 26). Although we attempted to eliminate some studies, the results did not change, and several additional studies are ongoing and we look forward to their inclusion in the meta-analysis when available.

Based on the above-mentioned limitations, the results of this study should be interpreted with caution. In particular, the efficacy of NCT cannot be emphasized for all LARC (19). According to the ESMO Guideline Opinion (36), different treatment regimens should be adopted according to the risk stratification of stage II/III rectal cancer. We believe that the heterogeneity of the studies in this meta-analysis was partly due to the lack of risk stratification of the samples. Therefore, a separate study of neoadjuvant chemotherapy according to EMVI, CRM, anal distance, and T stage should be considered in the future. Although several studies have analyzed these risk strata (13, 25, 27, 28, 30, 55), a meta-analysis was not possible due to lack of data. The results of the PROSPECT series (56–58) published recently are very important. However, of the 585 patients in the NCT group, 53 (9.1%) were converted to Lc-NCRT because of dissatisfaction with NCT. This selective NCT study did not provide results for patients with NCT alone. Therefore, it was not included in this meta-analysis. It is undeniable that the design of selective NCT has, to some extent, prevented some patients who do not respond well to NCT from becoming victims of clinical trials. Therefore, the study design of selective radiotherapy is more ethical and worthy of reference for future clinical research. Recently, neoadjuvant chemoradiotherapy combined with immunotherapy has become popular. Radiotherapy may induce the aggregation of tumor-infiltrating lymphocytes and the expression of PDL-1 in tumor tissues, change the tumor microenvironment, and increase the sensitivity of tumors to immunotherapy in rectal cancer patients with proficient mismatch repair (pMMR) (59, 60). In the TORCH study, immunotherapy combined with total neoadjuvant therapy (iTNT) increased the CR rate to more than 50%, which was significantly higher than that of neoadjuvant therapy alone. Among them, the CR rate of the population that received radiotherapy first was higher than that of the population that received immunotherapy first, which may be due to the increased sensitivity of radiotherapy to immunotherapy (61). More abscopal effects have been observed in clinical studies of radiotherapy combined with immunotherapy, which is considered as strong evidence that radiotherapy stimulates anti-tumor immune responses (62, 63). Therefore, even if some low-risk patients benefit from NCT, some patients with lower tumor location still need additional radiotherapy or even total neoadjuvant therapy combined with immunotherapy to achieve anal preservation with a watch-and-wait approach (64).

In conclusion, compared with Lc-NCRT, NCT has comparable long-term oncological outcomes and controllable safety and is superior to Lc-NCRT in terms of surgical complications and sphincter preservation. Due to limited data, the results still need to be treated with caution and the strategy of selective radiotherapy should be adopted in clinical practice, especially for patients with an unsatisfactory tumor response to NCT. How to accurately select risk subgroups suitable for NCT is one of the future research directions.

## Data Availability

The original contributions presented in the study are included in the article/supplementary material. Further inquiries can be directed to the corresponding authors.
